# Supplementary material for: Novel Insights Into N-Glycan Fucosylation and Core Xylosylation in C. reinhardtii
Source: Front Plant Sci. 2020 Jan 15;10:1686. doi: 10.3389/fpls.2019.01686 (PMC6974686; doi:10.3389/fpls.2019.01686)
Supplement: Supplementary file 1 [file Image_1.pdf]

genes affected by insertional cassettes

fucT		x						x		x	x	x	
xylT1A				x		x	x	x	x	x	x	x	
xylT1B					x		x		x		x	x	
man1A			x			x			x	x		x	H_2O

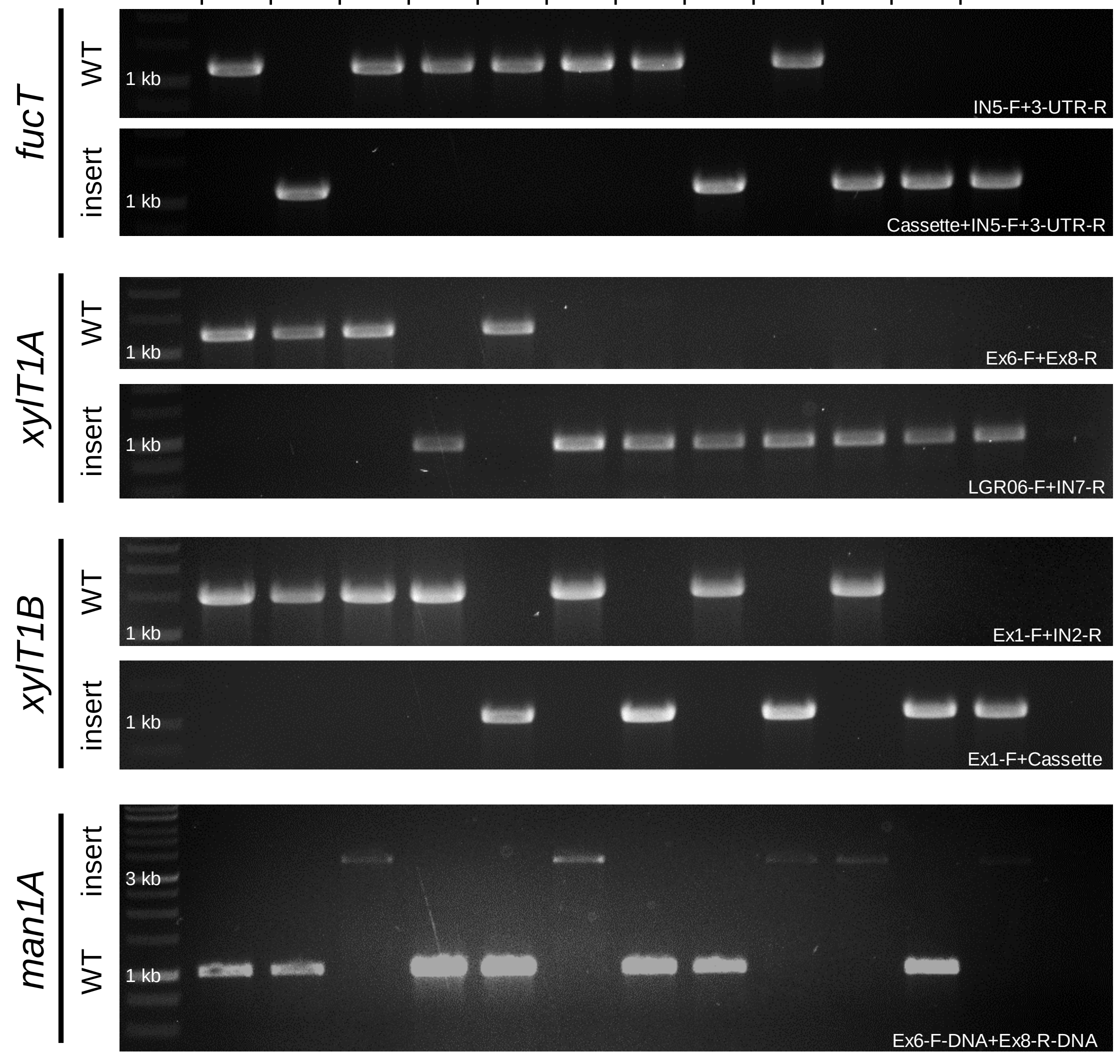

Supplemental Figure 1. Verification of *aphVIII* cassettes in respective genes of IM strains analyzed.

Primer pairs indicated at the right side were used in standard PCR reactions to test, whether genomic WT like regions or insertions of the DNA cassette encoding for the *aphVIII* gene, thus disrupting the coding sequence, are found at the respective sites. 1 % agarose gels stained employing MidoriGreen.
