# Supplementary material for: Novel Insights Into N-Glycan Fucosylation and Core Xylosylation in C. reinhardtii
Source: Front Plant Sci. 2020 Jan 15;10:1686. doi: 10.3389/fpls.2019.01686 (PMC6974686; doi:10.3389/fpls.2019.01686)
Supplement: Supplementary file 2 [file Image_2.pdf]

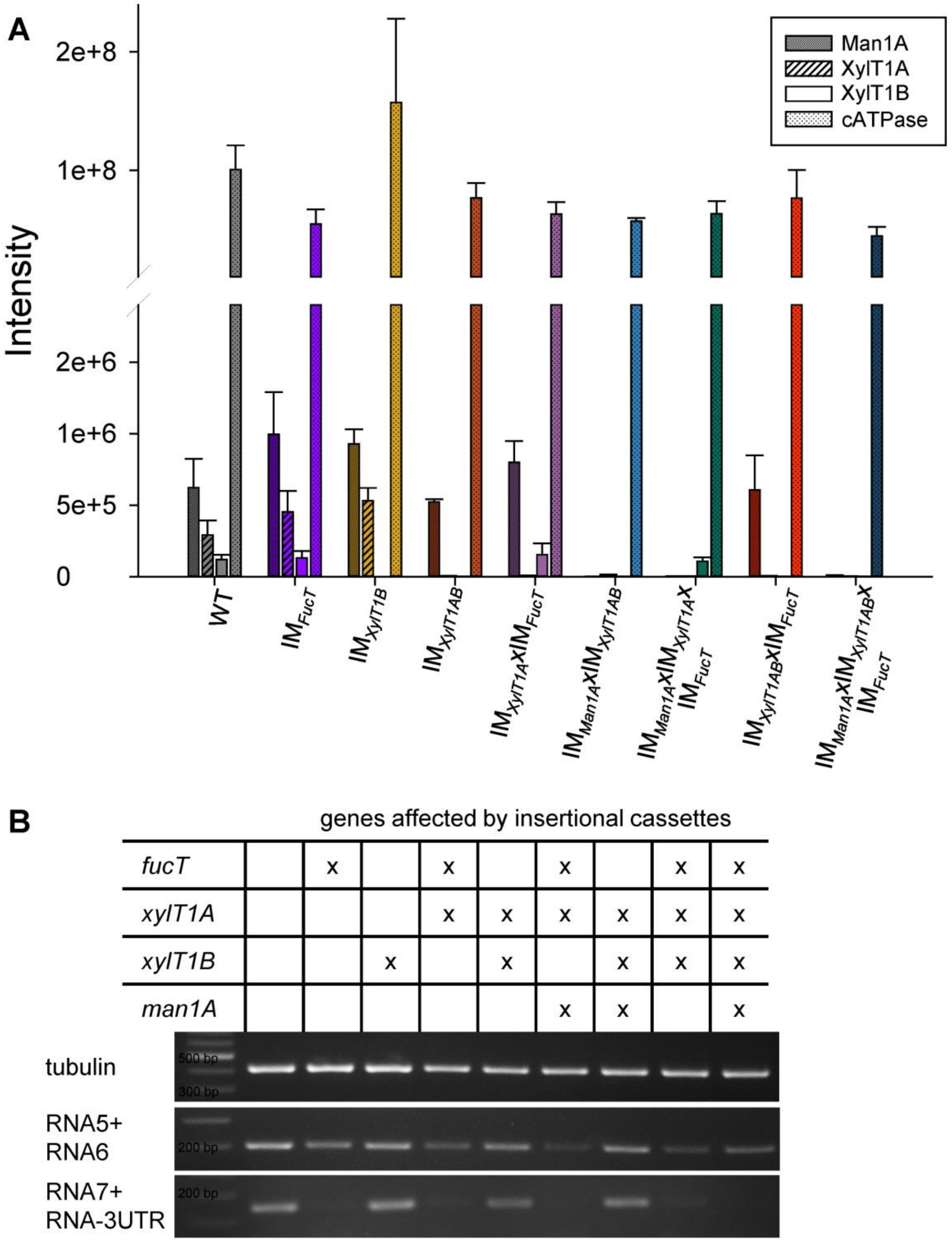

Supplemental Figure 2. PRM and cDNA reveal knockdown of affected enzymes in IM strains.

A, Parallel Reaction Monitoring (PRM) analyses assessing protein levels of enzymes encoded by genes disrupted due to insertional mutagenesis (Man1A, XylT1A and XylT1B) reveal signals below the detection limit. Error bars represent standard deviation between two (including a label swap) biological replicates for PRM results. B, Since no peptide of FucT was reliably detectable in WT, cDNA analysis was performed for FucT. Primer pairs binding prior (RNA5+RNA6) and after the insertion site (RNA7+RNA-3UTR) indicate a knockdown of FucT in the corresponding IM strains. Primer sequences can be found in Supplemental Table 2.
