# Supplementary material for: Novel Insights Into N-Glycan Fucosylation and Core Xylosylation in C. reinhardtii
Source: Front Plant Sci. 2020 Jan 15;10:1686. doi: 10.3389/fpls.2019.01686 (PMC6974686; doi:10.3389/fpls.2019.01686)
Supplement: Supplementary file 3 [file Image_3.pdf]

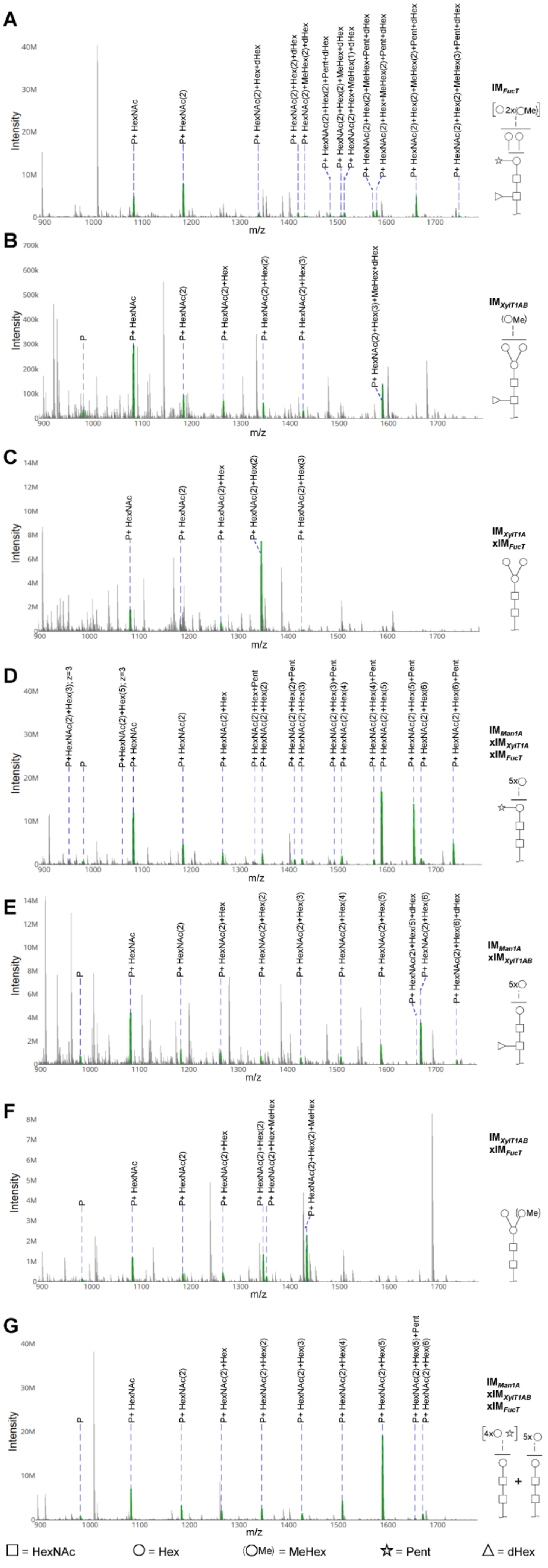
A
B
C
D
E
F
G
Intensity
m/z
P+ HexNAc
P+ HexNAc(2)
P+ HexNAc(2)+Hex
P+ HexNAc(2)+Hex(2)
P+ HexNAc(2)+Hex(3)
P+ HexNAc(2)+Hex+dHex
P+ HexNAc(2)+Hex(2)+dHex
P+ HexNAc(2)+MeHex(2)+dHex
P+ HexNAc(2)+Hex(2)+Pent+dHex
P+ HexNAc(2)+Hex(2)+MeHex+dHex
P+ HexNAc(2)+Hex+MeHex(1)+dHex
P+ HexNAc(2)+Hex(2)+MeHex+Pent+dHex
P+ HexNAc(2)+Hex+MeHex(2)+Pent+dHex
P+ HexNAc(2)+Hex(2)+MeHex(2)+Pent+dHex
P+ HexNAc(2)+Hex(2)+MeHex(3)+Pent+dHex
P+ HexNAc(2)+Hex(3)+MeHex+dHex
P+HexNAc(2)+Hex(3); z=3
P+HexNAc(2)+Hex(5); z=3
P+ HexNAc(2)+Hex+Pent
P+ HexNAc(2)+Hex(2)+Pent
P+ HexNAc(2)+Hex(3)+Pent
P+ HexNAc(2)+Hex(4)+Pent
P+ HexNAc(2)+Hex(5)+Pent
P+ HexNAc(2)+Hex(6)+Pent
P+ HexNAc(2)+Hex(4)
P+ HexNAc(2)+Hex(5)
P+ HexNAc(2)+Hex(6)
P+ HexNAc(2)+Hex(5)+dHex
P+ HexNAc(2)+Hex(6)+dHex
P+ HexNAc(2)+Hex+MeHex
P+ HexNAc(2)+Hex(2)+MeHex
P
IM FucT
IM XylT1AB
IM XylT1A
xIM FucT
IM Man1A
xIM XylT1A
xIM XylT1AB
2x
5x
4x
Me
= HexNAc
= Hex
= MeHex
= Pent
= dHex

Supplemental Figure 3. MS1 spectra assigned for *N*-glycan compositions using SugarPy.

MS1 spectra for representative *N*-glycan compositions identified in IM strains as being attached to the peptide ITYATTAAAVTNA**N**LSSYK (Asn residue carying HexNAc indicated by bold type letter). Please note that representation of WT was omitted as had been shown already in Schulze et al. 2018. Furthermore, also IM$_{XylT1B}$ is not presented, since its *N*-glycan composition is identical to WT. If not indicated otherwise, *N*-glycopeptides are present in charge state 2. Matched peaks are highlighted in green.
