# Supplementary material for: Novel Insights Into N-Glycan Fucosylation and Core Xylosylation in C. reinhardtii
Source: Front Plant Sci. 2020 Jan 15;10:1686. doi: 10.3389/fpls.2019.01686 (PMC6974686; doi:10.3389/fpls.2019.01686)
Supplement: Supplementary file 4 [file Image_4.pdf]

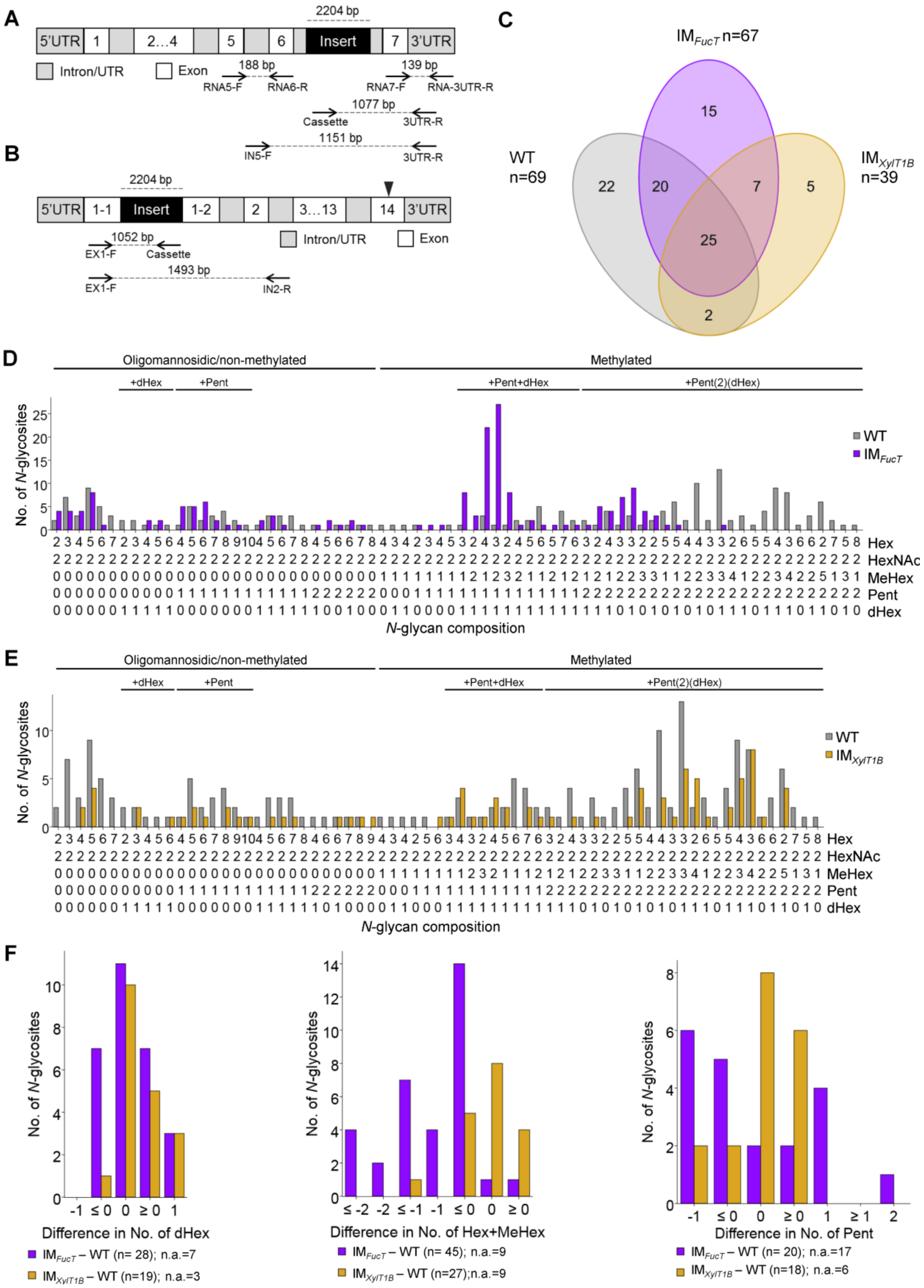

Supplemental Figure 4. *N*-glycan analysis of single IM strains reveals only minor changes.

Schematic representation of the genomic sequence of *fucT* (A) and *xylT1B* (B) (light grey: exon, dark grey: intron, black: insert). Primer pairs surrounding the cassettes were used to verify

WT like genomic regions whereas primer pairs involving the cassette primer verified an insertion of the *aphVIII* cassette in the respective genomic regions. The triangle indicate the position of peptides identified by PRM measurements. C, Venn diagram of *N*-glycosites for which the *N*-glycan composition could be determined. Number of all *N*-glycosites harboring the respective *N*-glycan composition are shown for WT (grey), IM_{FucT} (violet) (D) and IM_{XylT1B} (yellow) (E), respectively. The *N*-glycan complexity is increasing from left (oligomannosidic, not methylated) to right (decorated, methylated). *N*-glycan compositions were grouped according to the presence of Pent and/or dHex (optional for sugars written in parenthesis). All *N*-glycosites are taken into account. Peptide sequences and *N*-glycan compositions attached are listed in Supplemental Data 2. F, Differences in the number of dHex (left), *N*-glycan length, defined as the sum of Hex+MeHex (middle), and in the number of Pent (right) for *N*-glycosites found in both strains compared were calculated as depicted in the legend. *N*-glycosites carrying no dHex (left) or Pent (right) in both strains were excluded. The legends indicate the total number of *N*-glycosites compared. Some *N*-glycosites harboring multiple *N*-glycoforms could not be assigned to one of the categories (n.a.).
