# Supplementary material for: Novel Insights Into N-Glycan Fucosylation and Core Xylosylation in C. reinhardtii
Source: Front Plant Sci. 2020 Jan 15;10:1686. doi: 10.3389/fpls.2019.01686 (PMC6974686; doi:10.3389/fpls.2019.01686)
Supplement: Supplementary file 5 [file Image_5.pdf]

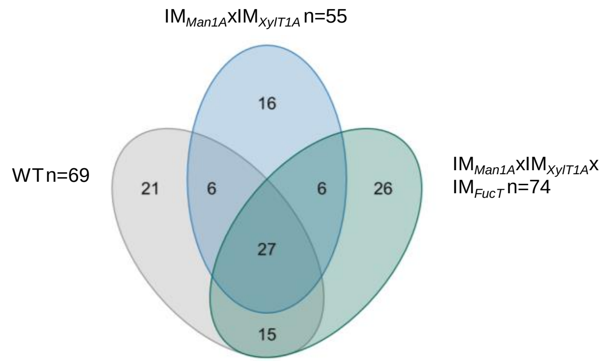

Supplemental Figure 5. Analysis of $IM_{Man1A}xIM_{XylT1A}xIM_{FucT}$

Venn diagram for *N*-glycosites for which the *N*-glycan composition could be determined in WT, $IM_{Man1A}xIM_{XylT1A}$ and $IM_{Man1A}xIM_{XylT1A}xIM_{FucT}$.
