# Supplementary material for: Novel Insights Into N-Glycan Fucosylation and Core Xylosylation in C. reinhardtii
Source: Front Plant Sci. 2020 Jan 15;10:1686. doi: 10.3389/fpls.2019.01686 (PMC6974686; doi:10.3389/fpls.2019.01686)
Supplement: Supplementary file 6 [file Image_6.pdf]

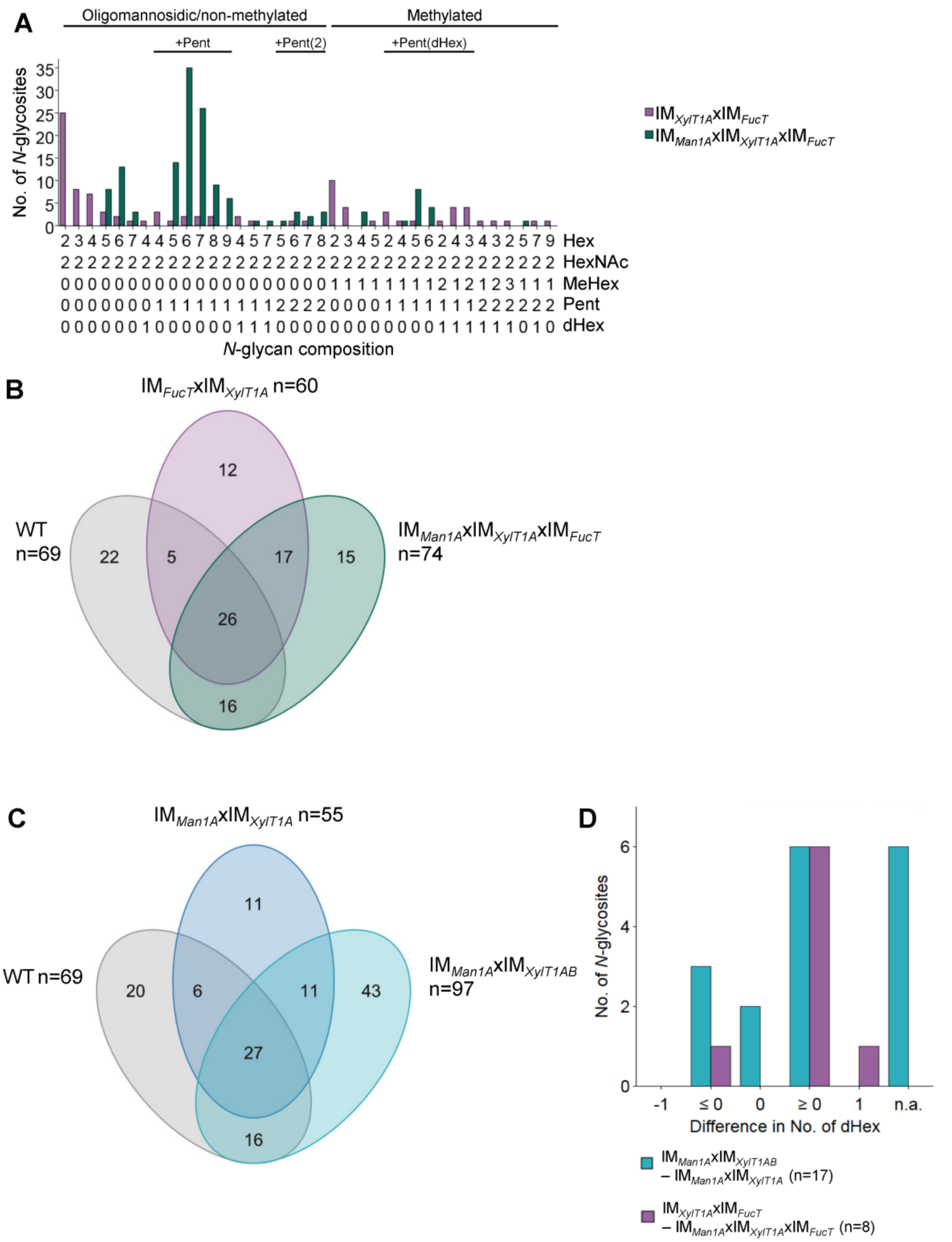

Supplemental Figure 6. Analysis of IM*Man1A*xIM*XylT1A*xIM*FucT*, IM*XylT1A*xIM*FucT* and IM*Man1A*xIM*XylT1AB*.

A, For all identified *N*-glycan compositions, the number of *N*-glycosites harboring this glycan is shown for IM*XylT1A*xIM*FucT* (light violet) and IM*Man1A*xIM*XylT1A*xIM*FucT* (green). The *N*-glycan complexity is increasing from left (oligomannosidic, not methylated) to right (decorated, methylated). *N*-glycan compositions were grouped according to the presence of Pent and/or dHex (optional for sugars written in parenthesis). All *N*-glycosites are taken into account. Peptide sequences and *N*-glycan compositions attached are listed in Supplemental Data 2. B, Venn diagram for *N*-glycosites for which the *N*-glycan composition could be determined for IM*XylT1A*xIM*FucT* and IM*Man1A*xIM*XylT1A*xIM*FucT*. C, Venn diagram for *N*-glycosites for which the

N-glycan composition could be determined for $IM_{Man1A}xIM_{XylT1A}$ and $IM_{Man1A}xIM_{XylT1AB}$. d, Differences in number of dHex for *N*-glycosites found in both strains were calculated. The legends indicate the total number of *N*-glycosites compared. Some *N*-glycosites harboring multiple *N*-glycoforms could not be assigned to one of the categories (n.a.).
