# Supplementary material for: Novel Insights Into N-Glycan Fucosylation and Core Xylosylation in C. reinhardtii
Source: Front Plant Sci. 2020 Jan 15;10:1686. doi: 10.3389/fpls.2019.01686 (PMC6974686; doi:10.3389/fpls.2019.01686)
Supplement: Supplementary file 9 [file Image_9.pdf]

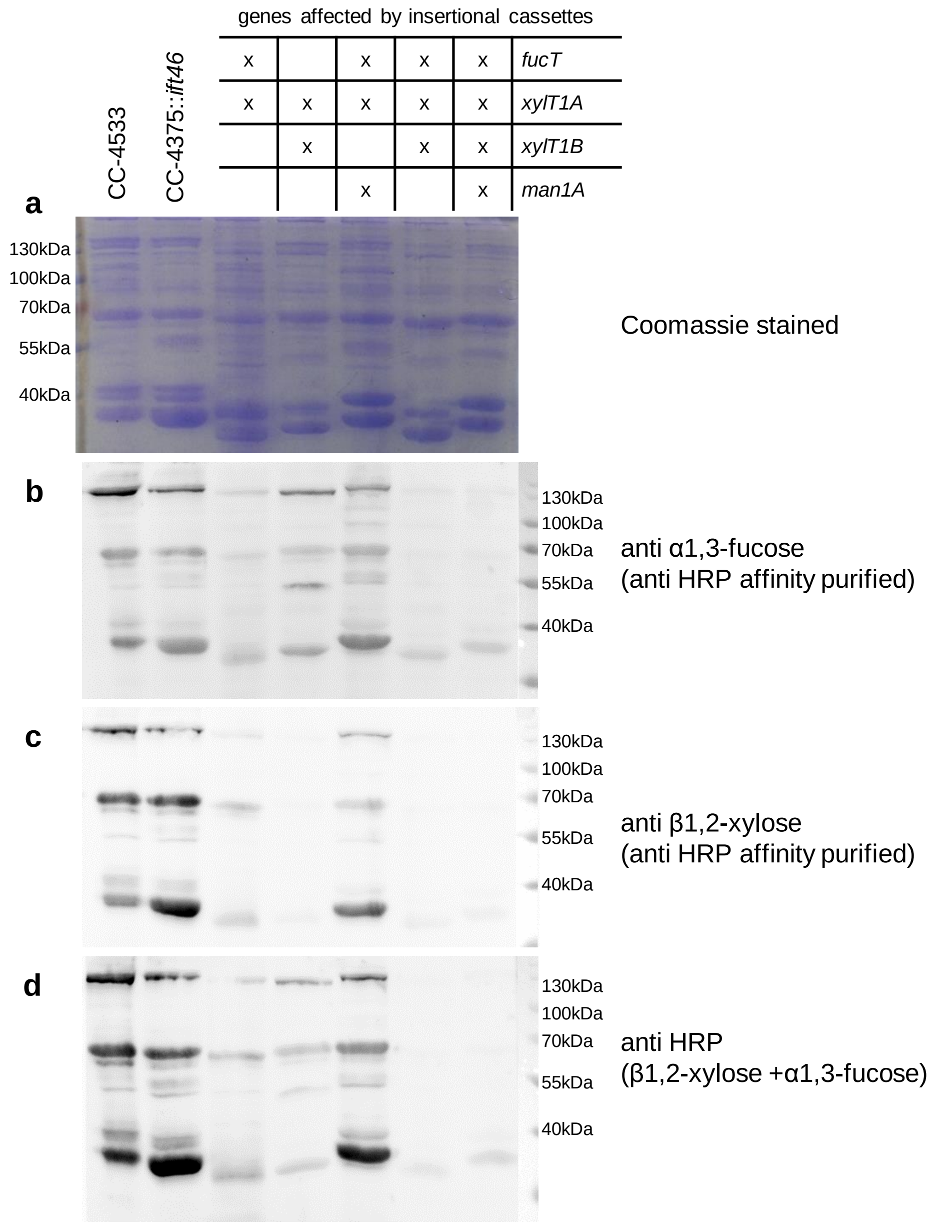

Supplemental Figure 9. Immunoblotting of residual IM confirms interdependence of core-xylosylation and fucosylation.

20 µg of SN proteins of selected IM strains were separated by SDS-PAGE and transferred to nitrocellulose membranes. Additionally, one SDS PAGE gel was stained as loading control using Coomassie Brilliant Blue G (A). Membranes were incubated in affinity purified HRP antibody binding to α-1,3-fucose (B) and β1,2-core xylose (C), respectively, as well as with the polyclonal HRP antibody (D). Please note the following considerations. In Figure 5, signal intensity of $IM_{Man1A}xIM_{XylT1A}xIM_{FucT}$ seems to be diminished, while here, in S9b, it seems enhanced. Those seemingly deviating results are on one hand attributed to signal intensities of strains loaded adjacent to the triple mutant. While strains loaded in Figure 5 result in very strong signals, the ones loaded in Supplemental Figure9b result in weak signals. When, in contrast, comparing signal intensities of $IM_{Man1A}xIM_{XylT1A}xIM_{FucT}$ to CC4533 (WT), the signal intensity ratios in both Figures are similar. Additionally, the loading control in Supplemental Figure9a reveals that slightly more protein had been loaded for $IM_{Man1A}xIM_{XylT1A}xIM_{FucT}$ additionally resulting in slightly elevated signals.
