# Supplementary material for: Novel Insights Into N-Glycan Fucosylation and Core Xylosylation in C. reinhardtii
Source: Front Plant Sci. 2020 Jan 15;10:1686. doi: 10.3389/fpls.2019.01686 (PMC6974686; doi:10.3389/fpls.2019.01686)
Supplement: Supplementary file 10 [file Table_1.pdf]

Supplemental Table 1. *N*-glycan compositions found in different strains analyzed for methylation degree as well as for Pent and dHex amount.

All *N*-glycosites identified in the strains listed were compared regarding methylation, Pent and dHex numbers attached. Boxes in dark grey highlight the loss of Pent upon excessive *N*-glycan trimming, due to a lack of XylT1-B action. *core Pent is defined as Pent, where the fragment peak Hex(1)HexNAc(2)Pent(1) was matched on MS1 level.

	**WT**	**IM$_{FucT}$**	**IM$_{XylT1\text{-}A}$x IM$_{FucT}$**	**IM$_{Man1A}$x IM$_{XylT1\text{-}A}$x IM$_{FucT}$**	**IM$_{Man1A}$x IM$_{XylT1\text{-}AB}$**	**IM$_{XylT1\text{-}AB}$x IM$_{FucT}$**	**IM$_{Man1A}$x IM$_{XylT1\text{-}AB}$x IM$_{FucT}$**
total No. of *N*-glycosites	194	184	122	146	249	225	238
N-glycosites with >1 MeHex	124	125	49	18	61	93	48
N-glycosites with 1 or 2 Pent	155	157	47	119	36	39	48
N-glycosites with core Pent *	>97	>97	>23	>65	>2	>9	>10
N-glycosites with dHex	123	116	18	3	63	5	2
N-glycosites with >1 MeHex / total No. of *N*-glycosites	63.9%	67.9%	40.2%	12.3%	24.5%	41.3%	20.2%
N-glycosites with 1 or 2 Pent / total No. of *N*-glycosites	79.9%	85.3%	38.5%	81.5%	14.5%	17.3%	20.2%
N-glycosites with 1 core Pent / total No. of *N*-glycosites	>50.0%	>52.7%	>18.9%	>44.5%	>8.0%	>4.0%	>4.2%
N-glycosites with dHex / total No. of *N*-glycosites	63.4%	63.0%	14.8%	2.1%	25.3%	2.2%	0.84%
