# Supplementary material for: Novel Insights Into N-Glycan Fucosylation and Core Xylosylation in C. reinhardtii
Source: Front Plant Sci. 2020 Jan 15;10:1686. doi: 10.3389/fpls.2019.01686 (PMC6974686; doi:10.3389/fpls.2019.01686)
Supplement: Supplementary file 11 [file Table_2.pdf]

Supplemental Table 2. Primer sequences used for verification PCRs and mRNA analysis.

Primer sequences used to analyze the presence or absence of an insert in the specific genomic region. Primers used for the verification of an insertion in the genomic regions encoding for Man1A and XylT1-A can be found in Schulze et al. 2018.

In addition, primer sequences used for mRNA analysis of FucT expression levels are given.

Primer name	Primer sequence	purpose
EX1-F	CTGTCGGTCGCGCAAATTGG	verification of insert in XylT1-B on DNA level
IN2-R	CCACCTCCACATTACGGCCT	
IN5-F	TGGAAGCCAGGACAACGCAC	verification of insert in FucT on DNA level
3UTR-R	CACGTCACAGCTTCCTGCCT	
Cassette	GCCCACGGTCAATTAGCCAC	verification of insert in XylT1-B and FucT on DNA level
RNA5-F	CAAGATGGAGCTGATCCGCG	relative quantification of mRNA level of FucT before insert
RNA6-R	AGCCCAGCCTGTTGTAGTCG	
RNA7-F	CTTCAGCACCTGCCTGTTCG	relative quantification of mRNA level of FucT following insert
RNA-3UTR-R	CTTCAGCACCTGCCTGTTCG	
tubulin-F	CACATCCAGGGTGGCCAG	relative quantification of tubulin mRNA (as housekeeping gene)
tubulin-R	CCTGGAAGCCCTGCAGGC	
