# Supplementary material for: Novel Insights Into N-Glycan Fucosylation and Core Xylosylation in C. reinhardtii
Source: Front Plant Sci. 2020 Jan 15;10:1686. doi: 10.3389/fpls.2019.01686 (PMC6974686; doi:10.3389/fpls.2019.01686)
Supplement: Supplementary file 12 [file Table_3.pdf]

Supplemental Table 3. List of mass spectrometry related parameters that used for the analysis intact *N*-glycopeptides using IS-CID and PRM measurements.

For HPLC, the mobile phases were composed of 0.1 % (v/v) formic acid in ultrapure water (A) and 80 % acetonitrile/0.08 % formic acid in ultrapure water (B). Percentage of buffer B is indicated as well as its increase (↑) or decrease (↓).

Method / Parameter	IS-CID for intact *N*-glycopeptides 50 cm column	PRM measurement for protein quantification
HPLC gradient	5 min 2.5 %	5 min 2.5 %
	40 min ↑ to 45 %	65 min ↑ to 18 %
	5 min ↑ to 99 %	50 min ↑ to 32 %
	20 min 99 %	5 min ↑ to 99 %
		20 min 99
	5 min ↓ to 2.5 %	5 min ↓ to 2.5 %
	30 min 2.5 %	30 min 2.5 %
IS-CID	80 eV	-
Mass tags	5 ppm	-
Excluded charges	unassigned, ≥5	-
Dynamic exclusion	15 s	-
Lock masses	off	best
MS1 scan range	600 – 3000 *m/z*	350-1600 *m/z*
MS1 AGC target	$3e^6$	$3e^6$
MS1 maximum injection time	100 ms	50 ms
MS2 scan range	dynamic, fixed first mass150 *m/z*	dynamic, fixed first mass 100 *m/z*
MS2 AGC target	$1e^5$	$5e^4$
MS2 maximum injection time	120 ms	240 ms
NCE	30	27
