# Supplementary material for: Novel Insights Into N-Glycan Fucosylation and Core Xylosylation in C. reinhardtii
Source: Front Plant Sci. 2020 Jan 15;10:1686. doi: 10.3389/fpls.2019.01686 (PMC6974686; doi:10.3389/fpls.2019.01686)
Supplement: Supplementary file 8 [file Image_8.pdf]

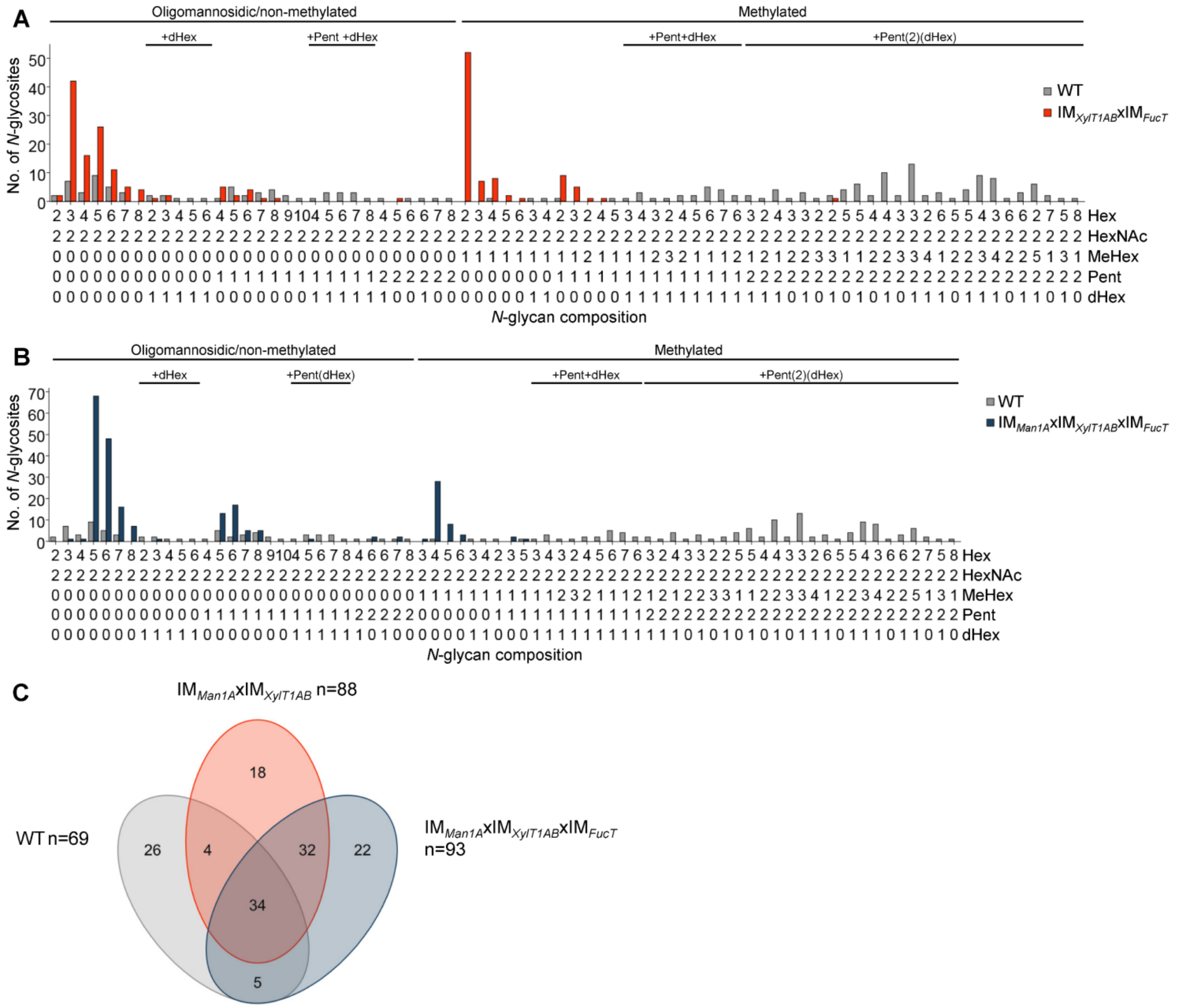

Supplemental Figure 8. Analysis of all *N*-glycosites in IM$_{XylT1AB}$xIM$_{FucT}$ and IM$_{Man1A}$xIM$_{XylT1AB}$xIM$_{FucT}$ reveals lack of *N*-glycan core modifications.

For all identified *N*-glycan compositions, the number of *N*-glycosites harboring this glycan is shown for WT (grey), IM$_{XylT1AB}$xIM$_{FucT}$ (orange) (A) and IM$_{Man1A}$xIM$_{XylT1AB}$xIM$_{FucT}$ (dark blue) (B), respectively. The *N*-glycan complexity is increasing from left (oligomannosidic, not methylated) to right (decorated, methylated). *N*-glycan compositions were grouped according to the presence of Pent and/or dHex (optional for sugars written in parenthesis). All *N*-glycosites are taken into account. Peptide sequences and *N*-glycan compositions attached are listed in Supplemental Data 2. C, Venn diagram for *N*-glycosites for which the *N*-glycan composition could be determined.
